# Interactive Curve-Linear Relationship Between Alteration of Carbohydrate Macromolecular Structure Traits in Hulless Barley (*Hordeum vulgare* L.) Grain and Nutrient Utilization, Biodegradation, and Bioavailability

**DOI:** 10.3390/ijms20061366

**Published:** 2019-03-18

**Authors:** Baoli Sun, Luciana L. Prates, Peiqiang Yu

**Affiliations:** 1 College of Animal Science, South China Agricultural University, Guangzhou 510642, China; baolisun@scau.edu.cn; 2 Ministry of Agriculture Strategic Research Chair Lab, College of Agricultural and Bioresources, University of Saskatchewan, Saskatoon, SK S7N5A8, Canada; lul614@mail.usask.ca; 3 College of Animal Science and Technology, Henan University of Animal Husbandry and Economy, Zhengzhou 450000, China

**Keywords:** alteration of carbohydrate traits, macromolecular, protein and carbohydrate fractions, ratio of amylose to amylopectin, β-glucan, biodegradation, bioenergy

## Abstract

The aim of this study was to reveal an interactive curve-linear relationship between altered carbohydrate macromolecular structure traits of hulless barley cultivars and nutrient utilization, biodegradation, as well as bioavailability. The cultivars had different carbohydrate macromolecular traits, including amylose (A), amylopectin (AP), and β-glucan contents, as well as their ratios (A:AP). The parameters assessed included: (1) chemical and nutrient profiles; (2) protein and carbohydrate sub-fractions partitioned by the Cornell Net Carbohydrate and Protein System (CNCPS); (3) total digestible nutrients (TDN) and energy values; and (4) in situ rumen degradation kinetics of nutrients and truly absorbed nutrient supply. The hulless barley samples were analyzed for starch (ST), crude protein (CP), total soluble crude protein (SCP), etc. The in situ incubation technique was performed to evaluate the degradation kinetics of the nutrients, as well as the effective degradability (ED) and bypass nutrient (B). Results showed that the carbohydrates (g/kg DM) had a cubic relationship (*p* < 0.05), with the A:AP ratio and β-glucan level; while the starch level presented a quadratic relationship (*p* < 0.05), with the A:AP ratio and cubic relationship (*p* < 0.05), with β-glucan level. The CP and SCP contents had a cubic relationship (*p* < 0.05) with the A:AP ratio and β-glucan level. The altered carbohydrate macromolecular traits were observed to have strongly curve-linear correlations with protein and carbohydrate fractions partitioned by CNCPS. For the in situ protein degradation kinetics, there was a quadratic effect of A:AP ratio on the rumen undegraded protein (RUP, g/kg DM) and a linear effect of β-glucan on the bypass protein (BCP, g/kg DM). The A:AP ratio and β-glucan levels had quadratic effects (*p* < 0.05) on BCP and EDCP. For ST degradation kinetics, the ST degradation rate (*K*_d_), BST and EDST showed cubic effects (*p* < 0.05) with A:AP ratio. The β-glucan level showed a cubic effect on EDST (g/kg DM) and a quadratic effect on BST (g/kg ST or g/kg DM) and EDST (g/kg DM). In conclusion, alteration of carbohydrate macromolecular traits in hulless barley significantly impacted nutrient utilization, metabolic characteristics, biodegradation, and bioavailability. Altered carbohydrate macromolecular traits curve-linearly affected the nutrient profiles, protein and carbohydrate fractions, total digestible nutrient, energy values, and in situ degradation kinetics.

## 1. Introduction

Barley (*Hordeum vulgare* L.) is one of the most important cereals cultivated in Canada, with approximately 8.78 million tons produced from 2015–2016 [1]. Although barley has been used in the malting industry, its high production and great metabolizable energy content (3.04 MCal/kg) [2] make this grain routinely used in the total mixed ration (TMR) in western Canada [3]. Thus, the majority (80%) of barley production is used domestically in feed production [4].

Barley mainly consists of a fibrous hull, pericarp, aleurone layer, endosperm, and germ [5]. The varieties of barley can be classified as hulled or hulless, in which hulless is defined as a spontaneous loss of hulls during harvest [6]. Overall, the presence of a hull decreases the nutritive value of the grain, because of the great fiber content, while hulless barley tends to show higher nutrient and metabolizable protein values [7,8]. As hulless barley does not requires pearling, there is a great retention of nutrient in the outer layer of the endosperm that makes hulless barley desirable as a food grain [9]. Moreover, barley grain is usually a great source of readily available energy for livestock, due to the presence of starch whose granules are mainly constituted of amylose and amylopectin [10,11]. Amylose is a linear molecule, constituted by glucose residues that are joined via α-1,4 linkages with very few α-1,6 linkages, while amylopectin contains linear chains at various degrees of polymerization, in which α-1,6 linkages join few glucose units that introduce branches to grow the amylopectin molecule [12]. The ratio of amylose to amylopectin is an important factor associated with the α-amylase activity of rumen microorganisms; consequently, the low ratio of amylose to amylopectin promotes a high starch degradation rate in barley grains [13]. Hulless barley cultivars have been developed by Crop Development Center (CDC, University of Saskatchewan) with altered carbohydrate macromolecular composition/traits, in which the proportion of amylose and amylopectin has been modified [8,14].

Barley grain contains β-glucan, a water-soluble fiber that is associated with the cell wall and correlated to the viscosity and gelatinization of the grain [15]. the ability of β-glucan to gelatinization in water may reduce the starch degradation rate in the rumen [15,16]. The modifications have been performed in order to improve the metabolic characteristics of barley. The presence of β-glucan can change the degradation of starch. However, less attention is given to β-glucan content in relation to degradation kinetics.

In previous studies, we evaluated the only linear effect of hulless barley with altered carbohydrate macromolecular structure traits on molecular structure features, chemical profiles, carbohydrate and protein sub-fractions, in situ rumen degradation kinetics, total metabolizable protein supply, and the molecular structure features [17,18,19]. We also evaluated the effect the curve-linear relationship between altered carbohydrate macromolecular structure traits, and the model predicted a truly absorbed protein supply and the molecular structures of hulless barley cultivars [20]. 

However, no research was found on the curve-linear relationship (e.g., quadratic or cubic response curve) between the altered carbohydrate macromolecular traits and chemical and nutrient profiles, nutrient utilization, and rumen degradation kinetics. The objective of this study was to evaluate the curve-linear relationship between hulless barley cultivars with altered amylose, amylopectin, β-glucan content with nutrient profiles, protein and carbohydrate sub-fractions, energy values, and in situ degradation characteristics of various nutrients.

## 2. Results and Discussion

### 2.1. Curve-Linear Relationship Between Altered Carbohydrate Macromolecular Traits and Chemical Profiles

Chemical and nutrient profiles showed large differences in the curve-linear relationship in hulless barley cultivars with altered carbohydrate (CHO) traits (Table 1). The dry matter of the hulless barley cultivars ranged from 906.4 to 916.9 g/kg and had no curve-linear relationship (*p* > 0.05) with carbohydrate macromolecular traits. Ash and ether extract (EE) had linear and quadratic responses (*p* < 0.05), respectively, to the amylose:amylopectin (A:AP) ratio and a linear response to β-glucan levels. The neutral detergent fiber (NDF) and non-structural carbohydrate (NSC) were quadratically related (*p* < 0.05) to the A:AP ratio and the β-glucan level. The acid detergent lignin (ADL) (g/kg dry matter (DM)) showed a quadratic relationship with the β-glucan level (*p* = 0.029), but the A:AP ratio has linear impact (*p* = 0.033) on ADL. The CHO (g/kg DM) showed a cubic response (*p* < 0.05) to the A:AP ratio and β-glucan level; however, the biological explanation for this response remains unclear. The starch content of hulless barley cultivars ranged from 487 to 630 g/kg of DM; it had a quadratic response (*p* < 0.05) to the A:AP ratio and a cubic response (*p* < 0.05) to the β-glucan level. Considering the protein profile, the crude protein (CP) and soluble crude protein (SCP) contents showed a cubic response (*p* < 0.05) to the A:AP ratio and the β-glucan level, while non-protein nitrogen (NPN) obtained a cubic response only to the β-glucan level. On the other hand, when the SCP was expressed as g/kg of CP, the cubic effect was observed only with the β-glucan level. Quadratic effect with the A:AP ratio, and to the β-glucan level, were obtained on neutral detergent insoluble crude protein (NDICP), expressed either as g/kg of the DM or g/kg of the CP. For acid detergent insoluble crude protein (ADICP) (g/kg DM), there was a quadratic effect (*p* < 0.05) with the A:AP ratio, while ADICP (g/kg CP) showed a quadratic response to the β-glucan level. 

During complex starch biosynthesis, variations in the granule compounds or compositions can modify the relationship between the A:AP ratio and other compounds, such as lipids and proteins [10]. The results in this study indicate the direct influence of the A:AP and β-glucan level on CHO and protein chemical profiles, where the greater β-glucan content in waxy and higher-than-normal amylose in starch barley grains affect the relationship between starch and β-glucan [21]. Furthermore, the β-glucan accounts for 3–7% DM in barley grains, and is mainly present in the endosperm [22,23]; consequently, the wide range of content, as well as the distribution throughout the grain, might contribute to the different responses on the chemical profile [15]. Although carbohydrates are the largest compounds in a cereal, the limitation in starch and NDF synthesis under specific weather conditions increases the protein proportion in the grain [24]. Likewise, there is a direct relationship between starch and protein contents, in which up to 40–50% of the total protein accounts for the protein matrix that surrounds the starch granules [25]. In addition, the starch is associated to protein in a heteromeric protein complex, which is affected by several factors that change the relationship between the protein profile and A:AP ratio [12]. Thus, it was expected that different responses between the protein profile and A:AP ratio, and between the protein profile and β-glucan level, would be provided from the complex interactions among the nutrients in the barley grain.

### 2.2. Curve-Linear Relationship Between Altered Carbohydrate Macromolecular Traits and Protein and Carbohydrate Fractions

Altered carbohydrate macromolecular traits among hulless barley cultivars were observed to have strongly curve-linear correlations with protein and CHO fractions partitioned by the Cornell Net Carbohydrate and Protein System (CNCPS) model (Table 2). The results of protein fracthe tions expressed in g/kg of CP showed linear responses (*p* < 0.05) of PB1, PB2, and PB3 fractions to the A:AP ratio. Regarding the β-glucan level, PB1 showed a linear response (*p* = 0.029), while a quadratic response was obtained for PB2 (*p* = 0.030) and PB3 (*p* = 0.044). The PC fraction had a quadratic effect (*p* = 0.046) with the β-glucan level. For the carbohydrate fractions expressed as g/kg of CHO, the CA and CB2 fractions presented a quadratic response to A:AP ratio, while a cubic effect was obtained for CB1, and a linear effect for the CC fraction. The β-glucan level presented cubic effects with CA and CB1 and quadratic effects with CB2 and CC. 

The A:AP ratio as well as the β-glucan level had great effects on the protein and CHO fractions. The CNCPS is an important tool to optimize the use of plant-based food/feed based on rumen function, microbial growth, digestion, and passage, and to model animals’ physiological states [26,27]. Although it is a mechanistic mathematical model, the A:AP ratios and β-glucan levels presented effective relationships with the fractions, and the particularities in the carbohydrates compounds and/or structures should be considered. This influence could be verified by the relationships obtained between the A:AP ratios, β-glucan levels, and degradable nutrients in the rumen. In the rumen degradation results, the effects were mainly cubic or quadratic, while the effects were linear in majority for the CNCPS sub-fractions. This difference might be related to the direct action of the microorganism on the substrates in the rumen degradation.

### 2.3. Curve-Linear Relationship Between Altered Carbohydrate Macromolecular Traits and Total Digestible Nutrients (TDN), Energy Values

The results related to the orthogonal polynomial response of truly digestible nutrients and energy values to altered CHO traits are shown in Table 2. The truly digestible non-fiber carbohydrate (tdNFC), truly digestible neutral detergent fiber (tdNDF), and truly digestible fatty acid (tdFA) contents presented quadratic responses (*p* < 0.05), while truly digestible crude protein (tdCP) content had a cubic response (*p* = 0.011) to the A:AP ratio. Regarding the β-glucan level, tdNFC (*p* = 0.014) and tdCP (*p* < 0.001) presented cubic responses, while a quadratic effect was obtained for tdNDF (*p* = 0.002), and a linear effect was obtained for tdFA (*p* = 0.005). The digestible energy at maintenance level (DE_1×_) was linearly related (*p* = 0.012) to the ratio of A:AP and quadratically related (*p* < 0.05) to the β-glucan level. The A:AP ratio presented a quadratic relationship, with digestible energy at a production level (DE_p3×_) (*p* = 0.016)_,_ : metabolizable energy at a production level (ME_p3×_) (*p* = 0.038), and net energy for lactation at a production level (NE_Lp3×_) (*p* = 0.004) and linear relationships between metabolizable energy (ME) (*p* = 0.017), : net energy for maintenance level (NE_m_) (*p* = 0.011), and net energy for growth level (NE_g_) (*p* = 0.036). The β-glucan level had cubic effects on DE_p3×_ (*p* = 0.004)_,_ ME_p3×_ (*p* = 0.011), and NE_Lp3×_ (*p* = 0.002); quadratic effects on DE_1×_ (*p* = 0.034) and NE_m_ (*p* = 0.043); and a linear relationship with NE_g_ (*p* = 0.037).

### 2.4. Curve-Linear Relationship Between Altered Carbohydrate Macromolecular Traits and Ruminal Degradation Kinetics of Various Nutrients

The curve-linear relationship between the ruminal degradation kinetics of DM, CP, NDF, starch (ST), and CHO in hulless barley varieties with altered CHO traits are shown in Table 3. Regarding the in situ rumen DM degradation, the *K*_d_ showed a cubic response (*p* < 0.001) and a quadratic response (*p* = 0.030) to the β-glucan level. The altered A:AP ratio and β-glucan level had quadratic effects on the percentage of rumen bypass dry matter (BDM) and effective degradability of dry matter (EDDM). For the CP degradation kinetics, the undegradable fraction (U) and effective degradability of crude protein (EDCP) (g/kg DM) presented cubic responses (*p* < 0.05) to the A:AP ratio and β-glucan levels. There was quadratic effect of the A:AP ratio on undegradable crude protein (RUP) (g/kg DM) and a linear effect of the β-glucan level on bypass of crude protein (BCP) (g/kg DM). The A:AP ratio and β-glucan level showed a quadratic effect (*p* < 0.05) on BCP expressed as g/kg of CP, as well as on EDCP (g/kg CP). Considering the NDF rumen degradation kinetics, the degradation rate of D fraction (*K*_d_) linearly changed (*p* < 0.05) with the β-glucan level. The rumen bypass NDF (BNDF) and effective degradability of NDF (EDNDF) (% NDF) were found to have linear responses (*p* < 0.05) to the ratio of A:AP and quadratic responses (*p* < 0.05) to the β-glucan level. There was a cubic effect (*p* = 0.008) on the A:AP ratio and a quadratic effect (*p* < 0.01) on EDNDF (g/kg DM). For the ST rumen degradation kinetics, the *K*_d_ of starch rumen degradation, the rumen bypass starch (BST) and effective degradability of starch (EDST) showed cubic responses (*P* < 0.05) to the A:AP ratio. The β-glucan level showed a cubic effect on the EDST (g/kg DM; *p* = 0.002) and quadratic effects on the *K*_d_ of starch rumen degradation (*p* < 0.001), soluble and degradable fractions (*p* = 0.032), BST (g/kg ST or g/kg DM), and EDST (g/kg DM; *p* < 0.001). For the rumen degradation kinetics of CHO, there were cubic effects of the A:AP ratio on *K*_d_ (*p* = 0.002), the undegradable fraction (*p* = 0.033), bypass carbohydrate (BCHO) (g/kg CHO or g/kg DM; *p* < 0.05), and effective degradability of carbohydrate (EDCHO) (g/kg CHO; *p* = 0.016). Regarding the β-glucan level, there was a cubic effect on EDCHO (g/kg DM; *p* < 0.01), and a quadratic effect on *K*_d_ (*p* < 0.01), BCHO (g/kg CHO or g/kg DM; *p* < 0.01), and EDCHO (g/kg CHO; *p* < 0.001). 

Considering the rumen degradation of the nutrients, the effect of the A:AP ratio might be related to enzymatic activities involved in the hydrolysis process, in which the activity of amylolysis tends to increase when a subtract is available. However, there is a low rate of amylolysis that can restrain the starch digestion [16]. In addition, there are some interactions, such as those between amylose and lipids, that might affect the ruminal degradation of DM. Overall, β-glucan contains two or three (1-4)-linked units separated by a single (1-3)-linkage. The single (1-3)-linkage is responsible for the irregular structure of the molecule, accounting for the properties and structure of the β-glucan, that includes its solubility characteristics [15]. Thus, high levels of β-glucan level tend to decrease the DM intake and, consequently, affect the rumen degradation of DM. 

Hulless barley cultivars with higher amylose and β-glucan have lower starch degradation rates and lower EDCP, which may reduce the risk of rumen acidosis and increase the protein availability for intestinal digestion [17]. These results might be related to the intrinsic relationship between protein contents and carbohydrate compounds in hulless barley, where the organization of the starch granule is a complex process involving classes of enzymes [12]. Likewise, the rumen degradation of β-glucan is dependent on the β-glucanase activity of rumen microbiota that could lead to variation in the rumen degradation characteristic of CP [22]. 

The starch degradation in cereal seeds involves the action of several enzymes that are regulated from different pathways. These enzymes first access the stored substrates after the endosperm cell walls are degraded; consequently, the degradation of the cell wall polysaccharides, primarily β-glucans, is a rate-limiting step in the mobilization of energy storages in the seed [28]. In addition, the amylose and amylopectin can form double helices, which may in turn associate to form crystalline domains [10]. This crystallization might be involved in the formation of resistant starch, which is defined as a starch unavailable for absorption in the small intestine [16]. Thus, the A:AP ratio might affect the formation of resistant starch that changes the rumen degradation of starch.

## 3. Materials and Methods

### 3.1. Sample Collection and Preparation

Hulless barley cultivars and lines with altered amylose, amylopectin, and β-glucan macromolecular structures were developed by the Crop Development Centre (Dr. Aaron Beattie, CDC) at the University of Saskatchewan, Saskatoon, SK, Canada. The CDC Fibar, CDC Rattan, CDC McGwire and HB08302 were the hulless barley varieties used, according to their amylose, amylopectin and β-glucan levels (Table S1). All hulless barley cultivars were planted and grown at the Crop Research Field in Western Canada at the University of Saskatchewan. Grains of each variety were sampled from research field plots (*n* = 2) grown in 2008, 2009, and 2010, except for HB08302 (grown in 2009, 2010). The sample preparation was performed as described by Yang et al. [17]. Briefly, approximately one-kg of each sample was crushed using a Sven Roller Mill, with a gap of 0.203 mm (Apollo Machine and Products Ltd., Saskatoon, SK, Canada), at the Department of Agricultural Engineering (University of Saskatchewan, Saskatoon, SK, Canada). A sub-sample (100 g) of each rolled sample was ground using a Retsch SM 2000 (Retsch, Inc., Newtown, PA, USA), fit with a 0.5 mm screen for the analyses of total starch (ST), amylose, amylopectin, and β-glucan level. Another sub-sample (100 g) was ground using a Retsch SM 2000 (Retsch, Inc.), fit with a 1.0 mm screen to determine other chemical compositions.

### 3.2. Chemical Analysis

Samples ground with a 1.0 mm screen were analyzed for DM, organic matter (OM), ether extracts (EE), and CP, according to AOAC [29] methods. The neutral detergent fiber (NDF), acid detergent fiber (ADF), acid detergent lignin (ADL), total carbohydrate (CHO) and true protein were determined according to Van Soest et al. [30] and NRC [2]. Starch, amylose, amylopectin, β-glucan, non-protein nitrogen (NPN), total soluble crude protein (SCP), neutral detergent insoluble CP (NDICP) and non-structural carbohydrates (NSCs) were analyzed in accordance with the procedures described by Yang et al. [17] and Damiran and Yu [31].

### 3.3. Partitioning Protein and Carbohydrate Fractions 

The Cornell Net Carbohydrate and Protein System (CNCPS) is a useful tool for research development and feed formulation, in which the protein and carbohydrate from feeds are partitioned according to the rumen degradation characteristics [32]. Proteins and carbohydrates were fractionated by the CNCPS, as described by Van Amburgh et al. [27,33]. The protein content was partitioned into: a PA fraction that assumes a soluble fraction (non-protein nitrogen-NPN) that is rapidly degradable with an infinite degradation rate; a PB1 fraction, which is the soluble true protein and has a degradation rate of 130–100%/h; a PB2 fraction, which is the moderately degraded true protein and has a degradation rate of 3–20%/h; a PB3 fraction, which is the slowly degraded true protein (fiber-bound protein) and has a degradation rate of 0.05–2.0%/h; and a PC fraction, which is the unavailable protein. The feed carbohydrate content was partitioned into: a CA fraction as sugar, a CB1 fraction as starch; a CB2 fraction as soluble fiber; a CB3 fraction as available NDF; and a CC fraction as an unavailable carbohydrate fraction, calculated by lignin (CC = (Lignin × 2.4)/100) [34]. The degradation rate for CA, CB1, CB2, CB3 and CC was 300–500%/h; 20–40%/h, 20–40%/h, 4–9%/h and 0%/h, respectively. The rumen nutrient supply could be predicted using CNCPS [35]. 

### 3.4. Estimation of Total Digestible Nutrient and Energy Value

The total digestible nutrients (TDN) and estimated energy value contents for total digestible non-fiber carbohydrate (tdNFCs), total digestible crude protein (tdCP), total digestible NDF (tdNDF), and total digestible fatty acid (tdFA) were determined using an NRC summative approach [2]. The TDN at a maintenance level of intake (TDN_1×_), digestible energy at a maintenance level of intake (DE_1×_), digestible energy at a production level (DE_p3×_), metabolizable energy at a production level (ME_p3×_), and net energy for lactation at a production level (NE_Lp3×_) were also determined using an NRC summative approach from the dairy nutrient requirement (NRC, 2001). The net energy for maintenance (NE_m_) and net energy for growth (NE_g_) were determined using NRC [36]. 

### 3.5. In Situ Incubation Technique and Degradation Kinetics

The degradation kinetic parameters were determined using an in situ incubation technique. The detailed methods of the incubation are referenced in the previous publications [37,38,39]. Briefly, three non-lactating Holsteins equipped with rumen cannulae were individually housed and tied during the trial in stalls (9-m^2^) with concrete floors that were coated with rubber. The stalls were equipped with individual feeders and drinkers. The animals were fed twice daily at 08.00 h and 16.00 h and received approximately 15 kg/day of a TMR. This diet was formulated according to NRC [2] to attend to the maintenance requirement. Seven grams of each individually rolled sample were weighed into a pre-weighed and numbered nylon bag (10 × 20 cm) with a pore size of approximately 40 μm and a weight to bag surface area of 19 mg/cm^2^. Samples were incubated in the rumen for 0, 2, 4, 8, 12, and 24 h. Rumen incubations were performed according to the “gradual addition/all out” schedule [40]. The bag’s washing and drying procedures were performed accordingly [31,40]. Dry samples were pooled according to grain cultivar, years, plots, incubation time, and in situ run. The pooled samples were ground using a Retsch SM 2000 (Retsch, Inc.) fit with a 1.0 mm screen and stored at 20–22 °C for further chemical analysis. The degradation characteristics of CP, ST, NDF, and CHO were assessed using the first-order kinetics degradation model described by Ørskov and McDonald [41] and modified by Tamminga et al. [42]. The rumen undegradable (RU) or rumen bypass (B) values of nutrients on a percentage basis were calculated according to the NRC [2] and Tamminga et al. [42], respectively. The rumen undegradable protein (RUP) and rumen bypass protein (BCP) were calculated in terms of the Dutch model [42] and the NRC 2001 model [2]. 

The effective degradability contents of the nutrients were calculated as:

$$ED\;\left(\%\right)=S+D\times K_{d}/\left(K_{p}+K_{d}\right)$$

ED (g/kgDM)=A(g/kg)×ED(%),

where, ED is the effective degradability, A is the nutrient DM, CP, NDF, CHO, or ST, S is the soluble fraction (%), D is for the potentially degradable fractions (g/kg), $K_{d}$ is the degradation rate (h^−1^), and $K_{p}$ was the passage rate that was assumed as 6%/h. Additional details related to in situ calculation have been described by Zhang and Yu [43] and Yang et al. [14].

### 3.6. Statistical Analysis

Data analyses were performed using the PROC MIXED procedure of SAS 9.3 (SAS Institute, Inc., Cary, NC, USA). Orthogonal polynomial contrasts were used to determine linear, quadratic and cubic responses of measured nutrient parameters to the altered ratio of A:AP and the β-glucan level. The orthogonal contrasts technique can be used to obtain information, such as comparisons between groups of means, and/or specific residuals, from experimental data. Furthermore, the orthogonal contrasts can be applied on data from experiments in which there was not a definite structure [44]. 

Residuals were plotted against the predicted values to check the model assumptions regarding independence, homoscedasticity and normality of the errors. A data point was regarded as an outlier and removed from the database if the Studentized residual was outside the ±3.0 range.

## 4. Conclusions

It was concluded that the altered carbohydrate macromolecular traits in hulless barley had curve-linear effects on the nutrient utilization, biodegradation, and bioavailability. The changes of the carbohydrate macromolecular traits resulted in a highly curve-linear impact on chemical and nutrient profiles, the protein and carbohydrate sub-fractions partitioned by the CNCPS model, total digestible nutrients, energy values, and in situ rumen degradation of DM, CP, NDF, starch, and carbohydrates. This study revealed a high sensitivity of nutrient profiles, biodegradation, and bioavailability in ruminant systems to altered carbohydrate macromolecular traits in hulless barley.

## Figures and Tables

**Table 1:** Curve-linear response of chemical and nutrient profiles to altered carbohydrate macromolecular structure traits in Crop Development Center (CDC) hulless barley grain.

Item	Chemical and Nutrient Profile of Hulless Barley							Orthogonal Polynomial Contrast (*p* Value)					
								Ratio of A:AP			β-Glucan Level		
	*n*	Mean	STD	Range	Min	Max	CV	L	Q	C	L	Q	C
Basic chemical profile													
DM (g/kg)	11	912.4	3.7	10.5	906.4	916.9	0.4	0.998	0.099	0.925	0.151	0.391	0.535
Ash (g/kg DM)	11	21.6	2.0	7.1	18.3	25.4	9.2	0.037	0.012	0.219	0.005	0.424	0.064
EE (g/kg DM)	11	25.0	2.3	7.4	20.1	27.5	9.3	0.807	0.003	0.713	0.005	0.095	0.186
Carbohydrate profile													
NDF (g/kg DM)	11	114.1	13.3	44.7	99.4	144.1	11.6	0.002	0.001	0.086	0.045	<0.001	0.574
ADF (g/kg DM)	11	25.4	3.7	12.8	17.8	30.6	14.4	0.884	0.241	0.862	0.327	0.599	0.554
ADL (g/kg DM)	11	5.8	1.2	3.6	4.0	7.6	20.1	0.033	0.493	0.164	0.436	0.029	0.398
CHO (g/kg DM)	11	810.4	17.2	52.4	783.3	835.7	2.1	<0.001	<0.001	0.005	0.000	0.004	<0.001
NSC (g/kg CHO)	11	882.9	16.1	51.4	858.9	910.3	1.8	0.052	0.003	0.231	0.017	0.005	0.556
Starch (g/kg DM)	11	562.1	41.8	143.3	487.0	630.3	7.4	0.052	<0.001	0.448	<0.001	0.040	0.006
Protein profile													
CP (g/kg DM)	11	143.0	14.1	42.2	122.2	164.4	9.8	<0.001	<0.001	0.015	<0.001	0.004	<0.001
SCP (g/kg DM)	11	71.1	5.2	15.7	64.0	79.7	7.3	<0.001	<0.001	0.015	<0.001	0.001	<0.001
NPN (g/kg DM)	11	8.3	2.2	6.7	4.4	11.1	26.7	0.492	0.315	0.074	0.068	0.372	0.584
NDICP (g/kg DM)	11	19.3	4.6	14.1	14.8	28.9	23.7	0.040	0.332	0.400	0.891	0.039	0.297
ADICP (g/kg DM)	11	1.5	0. 9	2.7	0.4	3.1	61.2	0.218	0.078	0.629	0.238	0.081	0.943
SCP (g/kg CP)	11	498.8	18.2	51.1	473.5	524.6	3.6	0.009	0.093	0.391	0.086	0.201	0.007
NPN (g/kg CP)	11	57.2	16.1	57.1	29.2	86.3	28.1	0.349	0.789	0.029	0.097	0.117	0.353
NDICP (g/kg CP)	11	137.3	40.4	121.1	90.3	211.4	29.4	0.014	0.964	0.317	0.217	0.030	0.094
ADICP (g/kg CP)	11	10.7	6.5	17.3	3.3	20.6	60.4	0.094	0.113	0.431	0.476	0.046	0.683

DM: dry matter; CP: crude protein; NDF: neutral detergent fiber; ADF: acid detergent fiber; ADL: acid detergent lignin; ADICP: acid detergent insoluble crude protein; NDICP: neutral detergent insoluble crude protein; NPN: non-protein nitrogen; SCP: soluble crude protein; EE: ether extract; CHO: carbohydrate; NSC: non-structural carbohydrate; NPN: non-protein nitrogen. STD: Standard deviation; Min: Minimum; Max: Maximum; CV: Coefficient of variation; L: Linear; Q: quadratic; C: cubic.

**Table 2:** Curve-linear response of protein and CHO fractions partitioned by using the Cornell Net Carbohydrate and Protein System (CNCPS), and energy values to altered CHO macromolecular structure traits in CDC hulless barley grain.

Item	*n*	Mean	STD	Range	Min	Max	CV	Orthogonal Polynomial Contrast (*p* Value)					
								Ratio of A:AP			β-Glucan Level		
								L	Q	C	L	Q	C
Protein fractions													
PA (g/kg CP)	11	58.8	16.8	61.1	29.2	90.3	28.4	0.451	0.698	0.258	0.890	0.882	0.173
PB1 (g/kg CP)	11	439.9	17.2	55.9	408.7	464.6	3.9	0.007	0.080	0.730	0.029	0.072	0.013
PB2 (g/kg CP)	11	363.9	54.0	156.5	270.1	426.6	14.8	0.007	0.485	0.580	0.115	0.030	0.024
PB3 (g/kg CP)	11	126.6	35.7	106.4	84.4	190.8	28.2	0.017	0.697	0.356	0.163	0.044	0.090
PC (g/kg CP)	11	10.7	6.5	17.3	3.3	20.6	60.4	0.094	0.113	0.431	0.476	0.046	0.683
Carbohydrate fractions													
CA (g/kg CHO)	11	320.8	38.4	130.6	266.6	397.2	11.9	0.009	0.001	0.147	<0.001	0.860	0.007
CB1 (g/kg CHO)	11	562.1	41.8	143.3	487.0	630.3	7.4	<0.001	<0.001	<0.001	<0.001	<0.001	<0.001
CB2 (g/kg CHO)	11	100.0	15.7	49.1	78.3	127.4	15.7	0.024	0.004	0.163	0.035	0.003	0.816
CC (g/kg CHO)	11	17.1	3.6	11.7	11.4	23.1	21.2	0.022	0.778	0.126	0.215	0.024	0.283
Truly digestible nutrients													
TDN (g/kg DM)	11	892.0	5.9	16.6	887.1	903.7	0.6	0.933	0.325	0.939	0.398	0.668	0.674
tdNFC (g/kg DM)	11	729.4	24.4	76.5	698.8	775.3	3.3	0.100	<0.001	0.693	0.001	0.100	0.014
tdCP (g/kg DM)	11	142.4	14.1	42.4	121.6	164.0	9.9	<0.001	<0.001	0.011	<0.001	0.003	<0.001
tdNDF (g/kg DM)	11	56.5	8.8	27.8	45.5	73.3	15.6	0.010	0.005	0.116	0.072	0.002	0.826
tdFA (g/kg DM)	11	15.0	2.3	7.4	10.1	17.5	15.5	0.807	0.003	0.713	0.005	0.095	0.186
Energy values													
DE_1×_ (Mcal/kg)	11	3.94	0.02	0.06	3.91	3.97	0.63	0.012	0.337	0.157	0.047	0.034	0.086
DEp_3×_ (Mcal/kg)	11	3.18	0.02	0.04	3.16	3.20	0.51	0.001	0.016	0.104	0.003	0.008	0.004
MEp_3×_ (Mcal/kg)	11	2.76	0.02	0.04	2.74	2.78	0.62	0.001	0.038	0.055	0.004	0.008	0.011
NELp_3×_ (Mcal/kg)	11	1.75	0.01	0.04	1.73	1.77	0.68	0.001	0.004	0.218	0.001	0.022	0.002
ME (Mcal/kg)	11	3.23	0.02	0.06	3.20	3.26	0.69	0.017	0.100	0.257	0.025	0.091	0.059
NE_m_ (Mcal/kg)	11	2.22	0.02	0.04	2.2	2.24	0.80	0.011	0.161	0.159	0.026	0.043	0.065
NE_g_ (Mcal/kg)	11	1.53	0.01	0.04	1.51	1.55	0.96	0.036	0.118	0.305	0.037	0.164	0.107

PA: NPN fraction; PB1: rapidly degradable TP fraction; PB2: moderately degradable TP fraction; PB3: slowly degradable TP fraction; PC: unavailable protein; CA: rapidly degradable carbohydrate fraction; CB1: intermediately degradable carbohydrate fraction; CB2: slowly degradable carbohydrate fraction; CC: unavailable carbohydrate; tdNFC: truly digestible non-fiber carbohydrate; tdCP: truly digestible crude protein; tdNDF: truly digestible neutral detergent fiber; tdFA: truly digestible fatty acid; TDN: total digestible nutrient at maintenance level; DE_1×_: digestible energy at maintenance level; DE_p3×_: digestible energy at a production level; ME_p3×_: metabolizable energy at a production level; NE_Lp3×_: net energy for lactation at a production level; ME: metabolizable energy; NE_m_: net energy for maintenance level; NE_g_: net energy for growth level. STD: Standard deviation; Min: Minimum; Max: Maximum; CV: coefficient of variation. L: Linear; Q: quadratic; C: cubic.

**Table 3:** Curve-linear response of nutrient biodegradation and bioavailability to altered CHO macromolecular structure traits in CDC hulless barley grain.

Item	*n*	Mean	STD	Range	Min	Max	CV	Orthogonal Polynomial Contrast (*p* Value)					
								Ratio of A:AP			β-Glucan Level		
								L	Q	C	L	Q	C
In situ DM degradation													
*K*d (%/h)	22	12.78	3.10	11.18	8.38	19.56	24.2	<0.001	<0.001	<0.001	0.160	0.030	0.237
S (%)	22	4.66	2.50	8.77	0.94	9.26	53.6	0.374	0.085	0.926	0.167	0.077	0.838
D (%)	22	84.00	2.72	9.54	80.50	90.04	3.2	0.788	0.811	0.246	0.632	0.420	0.515
U (%)	22	11.34	2.73	9.95	6.31	16.26	24.0	0.452	0.157	0.260	0.630	0.104	0.532
BDM (g/kg DM)	22	388.9	55.69	191.2	300.9	492.1	14.3	0.022	0.006	0.792	0.054	0.005	0.432
EDDM (g/kg DM)	22	611.0	55.69	191.2	507.8	699.0	9.1	0.022	0.006	0.792	0.054	0.005	0.432
In situ CP degradation													
S (%)	22	6.44	5.66	16.04	0.00	16.04	87.9	0.510	0.398	0.179	0.818	0.125	0.483
D (%)	22	86.61	6.30	23.32	74.60	97.92	7.2	0.304	0.620	0.904	0.668	0.715	0.312
RUP (g/kg DM)	22	65.00	10.25	38.17	45.99	84.16	15.7	0.212	<0.001	0.577	0.001	0.216	0.081
BCP (g/kg DM)	22	72.15	11.38	42.37	51.05	93.42	15.7	0.212	<0.001	0.576	0.001	0.216	0.081
EDCP (g/kg CP)	22	545.0	60.1	194.8	450.9	645.7	11.0	0.016	0.003	0.117	0.188	<0.001	0.992
EDCP (g/kg DM)	22	77.97	12.05	43.67	61.37	105.0	15.1	<0.001	0.814	0.001	0.001	<0.001	0.021
In situ NDF degradation													
*K*d (%/h)	22	11.54	9.16	41.25	1.57	42.82	79.4	0.526	0.133	0.143	0.035	0.680	0.944
S (%)	22	8.01	8.17	27.08	0.00	27.08	101.9	0.286	0.694	0.129	0.474	0.116	0.727
D (%)	22	54.63	10.44	46.53	41.08	87.61	19.1	0.636	0.495	0.401	0.969	0.317	0.681
U (%)	22	37.36	11.28	57.43	0.00	57.43	30.1	0.693	0.770	0.765	0.623	0.783	0.853
BNDF (g/kg DM)	22	58.67	9.31	39.38	46.66	86.04	15.8	0.912	0.019	0.539	0.079	0.146	0.286
EDNDF (g/kg DM)	22	56.77	11.33	49.31	42.00	91.31	19.9	<0.001	0.007	0.008	0.954	<0.001	0.540
In situ starch degradation													
*K*d (%/h)	22	13.80	4.74	18.36	8.64	27.00	34.3	0.028	0.005	0.003	0.689	<0.001	0.154
S (%)	22	5.66	6.66	28.54	0.00	28.54	117.6	0.083	0.684	0.066	0.298	0.032	0.957
D (%)	22	94.34	6.66	28.54	71.46	100.0	7.0	0.083	0.684	0.066	0.298	0.032	0.957
BST (g/kg DM)	22	172.3	33.22	132.0	93.06	225.0	19.2	<0.001	0.007	<0.001	0.320	<0.001	0.374
EDST (g/kg DM)	22	389.7	56.01	192.5	297.5	490.0	14.3	0.064	<0.001	0.003	<0.001	<0.001	0.002
In situ CHO degradation													
*K*d (%/h)	22	14.68	4.93	19.06	8.77	27.83	33.6	0.034	0.010	0.002	0.961	<0.001	0.125
S (%)	22	5.85	5.13	19.80	0.00	19.80	87.7	0.148	0.793	0.139	0.375	0.080	0.986
D (%)	22	85.63	7.19	29.99	63.38	93.37	8.4	0.140	0.981	0.052	0.163	0.062	0.824
U (%)	22	8.52	2.90	12.63	4.19	16.82	34.0	0.312	0.585	0.033	0.070	0.152	0.558
BCHO (g/kg DM)	22	148.2	18.34	59.00	123.7	182.7	12.3	<0.001	<0.001	0.001	0.416	<0.001	0.993
EDCHO (g/kg DM)	22	527.9	44.77	170.3	432.9	603.3	8.4	0.084	<0.001	0.566	<0.001	<0.001	<0.001

Kd: degradation rate of D fraction (%/h); S: soluble fraction; D: potentially degradable fraction; U: undegradable fraction; BDM, BNDF, BST and BCHO: bypass DM, NDF, ST, CHO, respectively; BCP: bypass of CP (DVE/OEB system); RUP: undegradable crude protein (NRC, 2001 model); EDDM, EDCP, EDNDF, EDST, and EDCHO: effective degradability of DM, CP, NDF, ST, CHO, respectively. STD: Standard deviation; Min: Minimum; Max: Maximum; CV: coefficient of variation. L: Linear; Q: quadratic; C: cubic.

## Supplementary Materials

The following are available online at https://www.mdpi.com/1422-0067/20/6/1366/s1.
