# Supplementary material for: Interactive Curve-Linear Relationship Between Alteration of Carbohydrate Macromolecular Structure Traits in Hulless Barley (Hordeum vulgare L.) Grain and Nutrient Utilization, Biodegradation, and Bioavailability
Source: Int J Mol Sci. 2019 Mar 18;20(6):1366. doi: 10.3390/ijms20061366 (PMC6471004; doi:10.3390/ijms20061366)
Supplement: Supplementary file 1 [file ijms-20-01366-s001.pdf]

Suppl. Table 1. Alteration of Carbohydrate macromolecular structure traits and sampling years of four developed hulless barley cultivars which vary in macromoleculars of amylose, amylopectin and β-glucan levels.

Lines or variety with alteration of carbohydrate macromolecular structure traits	Samples replications	Macromolecular Amylose (g/kg starch)	Macromolecular Amylopectin (g/kg starch)	Macromolecular β-glucan (% DM)
CDC Fibar	Y1, Y2, Y3	0.0	1000.0	Very high
CDC Rattan	Y1, Y2, Y3	50.0	950.0	High
CDC McGwire	Y1, Y2, Y3	250.0	750.0	Normal
HB08302	Y1, Y2	400.0	600.0	High
